# Breakdown of Mucin as Barrier to Digestive Enzymes in the Ischemic Rat Small Intestine

**DOI:** 10.1371/journal.pone.0040087

**Published:** 2012-06-29

**Authors:** Marisol Chang, Tom Alsaigh, Erik B. Kistler, Geert W. Schmid-Schönbein

**Affiliations:** 1 Department of Bioengineering, University of California San Diego La Jolla, California, United States of America; 2 Department of Anesthesiology and Critical Care, University of California San Diego Medical Center, San Diego, California, United States of America; 3 Institute of Engineering in Medicine, University of California San Diego, La Jolla, California, United States of America; Charité, Campus Benjamin Franklin, Germany

## Abstract

Loss of integrity of the epithelial/mucosal barrier in the small intestine has been associated with different pathologies that originate and/or develop in the gastrointestinal tract. We showed recently that mucin, the main protein in the mucus layer, is disrupted during early periods of intestinal ischemia. This event is accompanied by entry of pancreatic digestive enzymes into the intestinal wall. We hypothesize that the mucin-containing mucus layer is the main barrier preventing digestive enzymes from contacting the epithelium. Mucin breakdown may render the epithelium accessible to pancreatic enzymes, causing its disruption and increased permeability. The objective of this study was to investigate the role of mucin as a protection for epithelial integrity and function. A rat model of 30 min splanchnic arterial occlusion (SAO) was used to study the degradation of two mucin isoforms (mucin 2 and 13) and two epithelial membrane proteins (E-cadherin and toll-like receptor 4, TLR4). In addition, the role of digestive enzymes in mucin breakdown was assessed in this model by luminal inhibition with acarbose, tranexamic acid, or nafamostat mesilate. Furthermore, the protective effect of the mucin layer against trypsin-mediated disruption of the intestinal epithelium was studied in vitro. Rats after SAO showed degradation of mucin 2 and fragmentation of mucin 13, which was not prevented by protease inhibition. Mucin breakdown was accompanied by increased intestinal permeability to FITC-dextran as well as degradation of E-cadherin and TLR4. Addition of mucin to intestinal epithelial cells in vitro protected against trypsin-mediated degradation of E-cadherin and TLR4 and reduced permeability of FITC-dextran across the monolayer. These results indicate that mucin plays an important role in the preservation of the mucosal barrier and that ischemia but not digestive enzymes disturbs mucin integrity, while digestive enzymes actively mediate epithelial cell disruption.

## Introduction

The intestinal epithelium covering the gastrointestinal tract consists of a monolayer of enterocytes covered by a mucus gel layer. Together these two layers provide a dynamic and regulated barrier allowing selective passage of luminal contents into the intestinal wall. Loss of the epithelial/mucus layer integrity is a common feature in gastrointestinal diseases [1], [2] and intestinal ischemia encountered in different forms of shock [3], [4], [5].

The mucus gel layer, which ranges in thickness from 50–300 µm [6], is a hydrated polymeric gel composed of carbohydrates, lipids and protein [7]. The major protein component of the mucus layer is mucin, which consists of several isoforms, both secreted and membrane associated. Mucin is believed to protect the epithelial surface of the small intestine from luminal digestive enzymes, abrasion by food particles, and pathogens by forming a barrier between the lumen and the intestinal epithelium [8], [9], [10], [11]. The epithelial cells also form a selective barrier to molecules found in the lumen; this barrier depends on the integrity of intercellular junctions and the extracellular plasma membrane proteins. Changes in the environment of epithelial cells make these molecules targets for proteolytic attack [12], cause disruption of cell structure components influencing intracellular signaling [13], [14], [15], and impair epithelial barrier function [16].

Intestinal epithelial cells express numerous membrane proteins on the plasma membrane whose fate after disruption of the mucin layer is uncertain. We have reported that E-cadherin, which plays a major role in maintaining the intercellular junctions between epithelial cells [17] is degraded during intestinal ischemia [3]. Conversely the fate of other membrane molecules, e.g. toll-like receptor 4 (TLR4), which is usually associated with infection and sepsis [18], [19] and recently has been linked to hemorrhagic shock and intestinal ischemia [20], [21], remains unknown. Since during ischemia disruption of mucin 2 (secreted) and mucin 13 (membrane bound) is accompanied by transport of digestive enzymes into the intestinal wall, we hypothesized that mucin is a barrier to luminal digestive enzymes under normal physiological conditions, as contact by digestive enzymes with the epithelium due to the absence or degradation of mucin results in receptor destruction and loss of epithelial cell integrity and function.

In this study we studied whether mucin disruption observed during intestinal ischemia is accompanied by impaired epithelial cell integrity and function. Using a rat model of intestinal ischemia by splanchnic arterial occlusion (SAO) we studied the fate of two mucin isoforms (mucin 2 and mucin 13) and two selected membrane proteins (E-cadherin and TLR4), as well as mucin 2 mRNA levels during and after the ischemic period. We also examined the effect of digestive enzymes on mucin and epithelial cell disruption by luminal amylase inhibition with acarbose and serine protease inhibition with tranexamic acid and nafamostat mesilate. Furthermore, we utilized rat intestinal epithelial cell cultures to demonstrate that addition of a mucin layer on the apical side protects epithelial cells against trypsin-mediated disruption.

**Figure 1:**
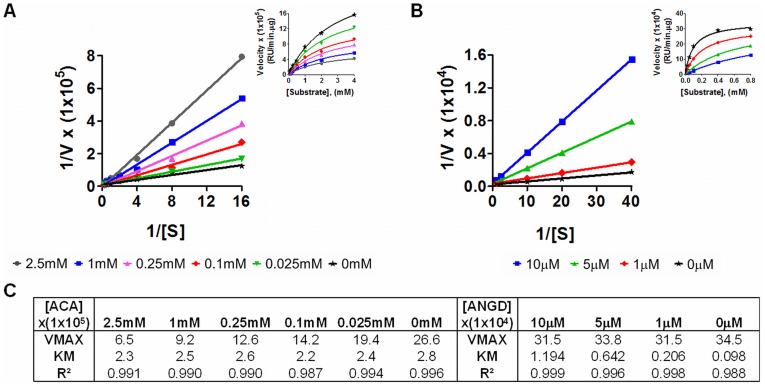
Inhibitory profile of acarbose and nafamostat mesilate. Lineweaver-Burk Plot with inset Michaelis-Menten plot of amylase activity with different concentrations of acarbose (**A**) and trypsin activity with different concentrations of nafamostat mesilate (**B**). Enzyme kinetic parameters, maximum enzyme velocity (V_max_, RFU/min.µg), Michaelis-Menten constant (K_m_, mM or µM) for amylase or trypsin with different concentrations of inhibitors calculated after non-linear regression (**C**).

## Materials and Methods

### Animal Groups and SAO Model

All animal protocols were reviewed and approved by the University of California San Diego Animal Subjects Committee. Male Wistar rats (300–350 g, Harlan Sprague Dawley Inc, Indianapolis, IN) were randomly assigned to one of five groups (n = 4 per group): a sham group (SHAM), and four groups subjected to 30 min splanchnic ischemia without enteral protease inhibition (SAO30) and with enteral amylase inhibition by acarbose (SAO30+ACA), protease inhibition by tranexamic acid (SAO30+TA) and nafamostat mesilate (ANGD, [6-amidino-2-naphthyl p-guanidinobenzoate dimethanesulfonate]) (SAO+ANGD). Rats were kept on solid food restriction for 12 hours prior to surgery with water ad libitum.

**Figure 2:**
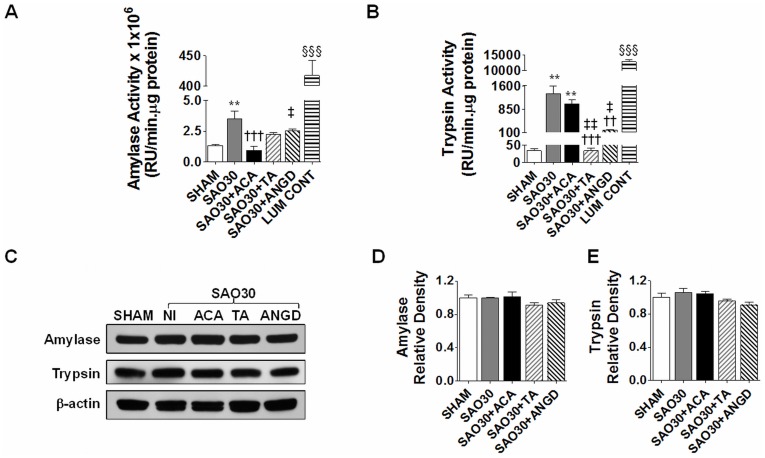
Amylase and trypsin activity in intestine homogenates is increased during SAO. Enzyme activity of amylase (**A**) and trypsin (**B**) in intestine homogenates of SHAM animals or animals subjected to SAO protocol with luminal inhibition using acarbose (ACA), tranexamic acid (TA) or nafamostat mesilate or without (NI). Activity of luminal contents of SHAM intestines for each enzyme is shown at the end of the graphs. Western blot for amylase, trypsin and β-actin in intestine homogenates of groups described above (**C**) with corresponding density levels measurements (**D, E**). Values are mean±SEM (n = 4)/group **P<0.001 compared to SHAM, †††P<0.0001 compared to SAO30, ‡P<0.05 and ‡‡P<0.001 compared to ACA, §§§ P<0.0001 compared to all the other groups.

After general anesthesia (Ketamine/Xylazine, 75 mg/kg Body Weight (BW)/20 mg/kg BW, intra-muscular) all groups were injected 0.9% saline (3 ml/100 g BW) into the lumen of the intestine either alone (SHAM or SAO30 groups) or mixed with one of three enzyme inhibitors (SAO30+ACA, SAO30+TA, or SAO30+ANGD groups). The concentration of the enzyme inhibitors was as follows: acarbose (0.5 mg/100 g BW) and tranexamic acid (0.1 g/100 g BW) (Sigma-Aldrich, St Louis, MO) or nafamostat mesilate (5 mg/100 g BW, Torii Pharmaceutical Co. Ltd., Tokyo, Japan). 30 min after saline or saline/inhibitor injection, the superior mesenteric and celiac arteries were isolated and occluded for 30 min for the ischemic groups or isolated without occlusion in the sham group. After 30 min SAO or sham surgery the animals were euthanized with Beuthanasia® 0.22 ml/kg BW, intra-venous.

**Figure 3:**
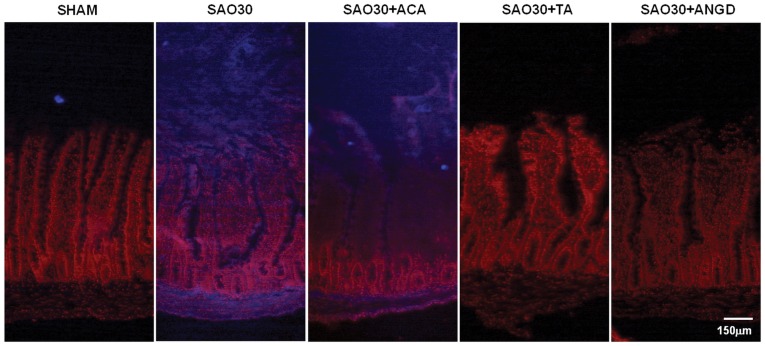
In situ zymography for trypsin in jejunal sections. Representative micrographs of trypsin activity as observed by fluorescence of specific substrate (blue), nuclei counterstaining with propidum iodine (red) in SHAM animals or animals subjected to SAO protocol with luminal inhibition with acarbose (ACA), tranexamic acid (TA) or nafamostat mesilate or without inhibitors (SAO30).

### Assessment of Intestinal Permeability In-Vivo

In separate experiments, rat jejunal sections were assayed for in vivo FITC-dextran transport (20 kDa, Sigma-Aldrich) from the lumen of the intestine into the intestinal wall. Groups were the same as described above with FITC-dextran (100 mg/ml) added to saline or saline/inhibitor solution prior to injection in the lumen of the intestine followed by 30 minutes ischemia or sham surgery and euthanasia.

### Tissue Cryosections

After euthanasia, jejunal sections (∼1 cm in length) were excised without removal of luminal contents and suspended in Tissue-Tek O.C.T. Compound (Sakura Finetek, Torrance, CA), snap frozen in isopentane/liquid nitrogen, and stored at −80°C for analysis. Cryosections (5 µm thickness) along the longitudinal axis of the villi were used throughout all experiments. Cryosections were fixed in 10% formalin solution and processed in a non-blinded fashion.

**Figure 4:**
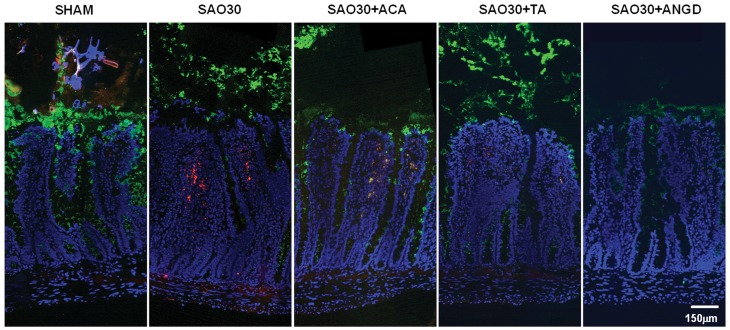
Tissue localization of mucin 2 and trypsin on jejunal sections. Representative immunostaining of mucin 2 (green), trypsin (red), with nuclei counterstaining (blue) for jejunal sections corresponding to SHAM animals or animals subjected to SAO protocol with luminal inhibition with acarbose (ACA), tranexamic acid (TA) or nafamostat mesilate or without inhibitors (SAO30).

### In-situ Tissue Zymography and Immunohistochemistry

In-situ zymography for trypsin activity in cryosections was assessed by measurement of fluorescence resulting from the proteolytic cleavage of the substrate (1 mM, Nα-benzoyl-L-arginine-7-amido-methylcoumarin hydrochloride; Sigma-Aldrich) as described elsewhere [22]. For immunohistochemical analysis, primary antibodies were diluted as followed: Trypsin 1∶300 and mucin2 1:200 (Santa Cruz Biotechnology, Santa Cruz, CA); the extracellular domain of TLR4 1:250 (Abcam, Cambridge, MA), and the intracellular domain of TLR4 1:250 (Invitrogen, Carlsbad, CA, USA). Secondary antibodies were as follows: FITC (Santa Cruz Biotechnology) and HRP (ImmPRESS, Vector Lab; Burlingame, CA). The slides were developed using 3, 3′-diaminodbenzidine substrate and counterstained with hematoxylin or DAPI (Vector Lab) or propidium iodine (Sigma-Aldrich). Slides were observed in a non-blinded fashion under an inverted microscope (20× and 60× objectives).

**Figure 5:**
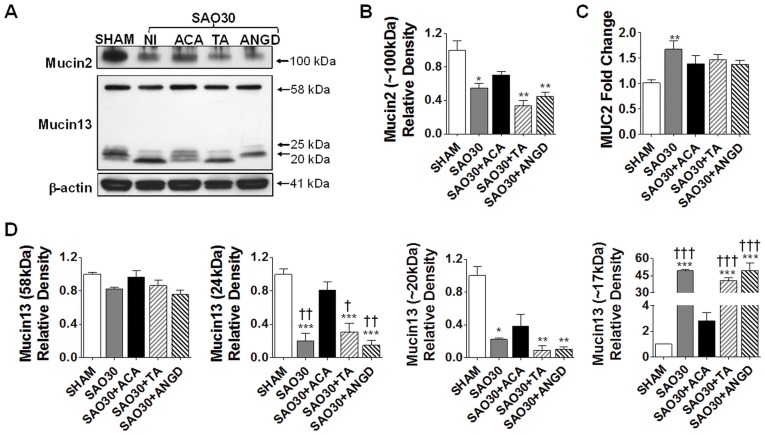
Mucin isoforms are degraded and MUC2 mRNA is up-regulated during intestinal ischemia. Western blot for mucin 2 and mucin 13 in jejunal homogenates of SHAM animals or animals subjected to SAO protocol with luminal inhibition with acarbose (ACA), tranexamic acid (TA) or nafamostat mesilate or without (NI) (**A**). Density levels measurement of mucin 2 (**B**) and mRNA expression of MUC2 (**C**). Mucin 13 density levels of different fragments (**D**). Values are mean±SEM (n = 4)/group **P<0.001 compared to SHAM, †††P<0.0001 compared to SAO30, ‡P<0.05 and ‡‡P<0.001 compared to ACA.

### Homogenates of Intestine and Luminal Contents

Jejunal segments were excised without removal of luminal contents. For enzyme activity measurements, segments of intestine were homogenized with CelLytic™ (Sigma-Aldrich) without addition of protease inhibitors. For Western blot assays the intestine segments were homogenized as above in the presence of protease inhibitors (5 mM EDTA, 5 mM N-Ethylmaleimide, 25 mM iodoacetamide, 5 mM benzamidine, 300 mM acarbose, 5 mM 6-aminocaproic acid, 1 mM protease inhibitor cocktail, (Sigma-Aldrich). In separate experiments the small intestine of sham animals was excised, and the luminal contents were flushed with 20 ml saline. Homogenates or luminal contents were centrifuged (16,000 g for 15 min at 4°C), the supernatant was collected and protein concentration was assessed with the bicinchoninic acid protein assay (Thermo Scientific).

### Enzyme Activity

Activity of intestine homogenates (100 µg of protein), luminal contents (50 µg of protein), or 50 µg/ml purified enzyme (porcine trypsin and amylase) was measured with substrates specific for trypsin and papain (50 µM, Nα-benzoyl-L-arginine-7-amido-methylcoumarin hydrochloride) [23], and amylase (4 mM, 2-Chloro-4-nitrophenyl-α-D-maltotrioside, Sigma-Aldrich) [24]. The initial rates of hydrolysis were measured by the fluorescent intensity of 7-amido-4-methylcoumarin, 380/460 nm (excitation/emission) or the absorbance of 2-Chloro-4-nitrophenol at 405 nm (SpectraMax Gemini XS, Molecular Devices, Sunnyvale, CA). The enzymatic inhibitory properties of acarbose and nafamostat mesilate were confirmed by incubation of amylase or trypsin with different concentrations of inhibitors for 30 minutes, followed by incubation with the specific substrates. The initial velocity of the reaction was expressed as the rate of change of Relative Units (RU) per µg of protein per minute. The data was fitted with a non-linear regression of the Michaelis-Menten or Lineweaver-Burk equations and kinetic constants (maximum enzyme velocity, V_max_, Michaelis-Menten constant, K_m_, and inhibitory constant, K_i_) were computed (GraphPad Prism, Graphpad Software, San Diego, CA).

### Quantitative PCR (qPCR)

Total-RNA was isolated from jejunal segments (SHAM (n = 6) and SAO30; n = 4 for SAO30+ACA, SAO30+TA and SAO30+ANGD) using RNeasy kit (Qiagen, Valencia, CA), and the total RNA was quantified by optical density. cDNA was generated (iScript cDNA synthesis kit, BioRad Laboratories, Richmond, CA) and qPCR reactions were performed (SYBR Green, Biorad). The primer sequences used for rat mucin 2 (MUC2) were designed from the fragment of *Rattus norvegicus* mucin mRNA (GI 506641): 5′-CAGAGTGCATCAGTGGCTGT-3′ (forward) and 5′-CCCGTCGAAGGTGATGTAGT-3′ (reverse). ß-actin was used as a reference gene, chosen and designed from the ß-actin mRNA (GI 42475962): 5′-AACTGGGACGATATGGAGAAGATTT-3′ (forward) and 5′-TGGGCACAGTGTGGGTGA-3′ (reverse). The efficiencies of the primers were determined and used for calculating the change in MUC2 mRNA expression relative to β-actin mRNA expression.

### Epithelial Cell Culture

IEC-18 cells (CRL-1589; ATCC, Manassas, VA), a cell line derived from the ileum of rat intestine, were grown in Dulbecco’s modified Eagle’s medium (DMEM) supplemented with 4 mM L-glutamine, 1.5 g/L sodium bicarbonate, 4.5 g/L glucose, 0.1 Unit/ml bovine insulin, 95%; and fetal bovine serum, 5%.

### Immunocytochemistry

IEC-18 cells were grown in 8-well culture slides (BD Biosciences, San Jose, CA) until 100% confluency was reached. On the day of the experiment the media was removed and cells were rinsed three times with PBS followed by incubation with serum free media (SFM) with or without porcine trypsin (5 µM, Sigma-Aldrich) for 60 min. Cells were fixed with 4% paraformaldehyde in PBS followed by blocking and permeabilization in 2% BSA in 0.1% PBS-T for 1 hr at room temperature. The slides were incubated overnight with primary antibody E-cadherin 1∶250 (Abcam) followed by incubation with FITC secondary antibody (Santa Cruz Biotechnology) and Alexa Fluor® 568 phalloidin (Invitrogen). Slides were observed using an inverted microscope (20× objective) using the appropriate fluorescent filters.

**Figure 6:**
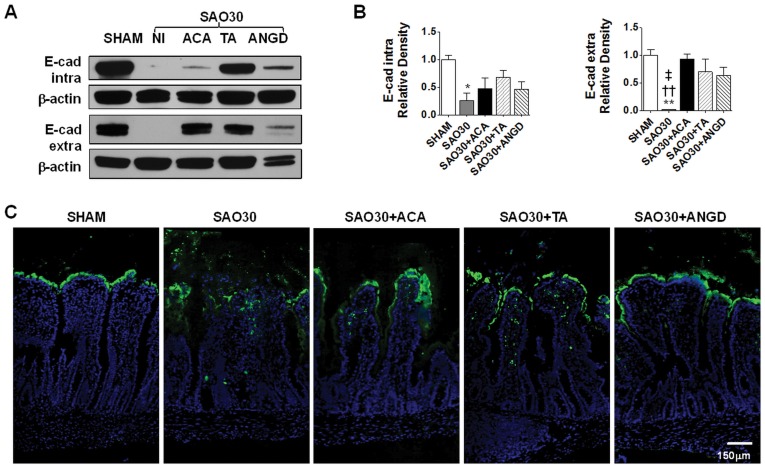
Epithelial barrier is disrupted during intestinal ischemia. Western blot for intra- and extra-cellular domains of E-cadherin in jejunal homogenates of SHAM animals or animals subjected to SAO protocol with luminal inhibition with acarbose (ACA), tranexamic acid (TA) or nafamostat mesilate or without (NI) (**A**) with corresponding density measurements. Values are mean±SEM (n = 4)/group * P<0.01 compared to sham, †† P<0.001 as compared to ACA, ‡ P<0.01 as compared to TA (**B**). Representative micrographs of jejunal sections for groups described above, showing localization of FITC-dextran (green) with nuclei counterstaining (blue) (**C**).

### Mucin Layer Addition to Epithelial Cells In-Vitro

IEC-18 cells were grown in 10 ml Transwell dishes (0.4-µm filter; Corning-Costar Corp) until 100% confluency. For the experiment the media was removed and cells were rinsed three times with phosphate buffered saline (PBS), the basolateral side was filled with SFM and the apical side was covered with or without a mucin film (∼400 µm, 10% in SFM, porcine stomach; Sigma-Aldrich, St. Louis, MO). SFM with or without 5 µM trypsin was carefully added to the apical side. Cells were incubated for 60 min at 37°C.

**Figure 7:**
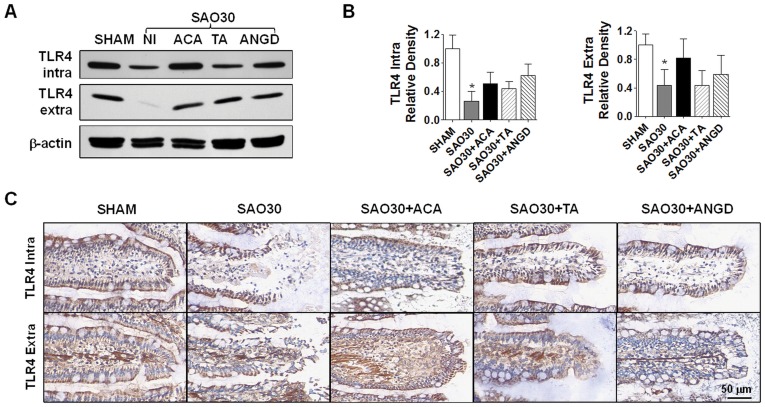
TLR4 is degraded during intestinal ischemia. Western blot for intra- and extra-cellular domains of TLR4 in jejunal homogenates of SHAM animals or animals subjected to SAO protocol with luminal inhibition with acarbose (ACA), tranexamic acid (TA) or nafamostat mesilate or without (NI) (**A**) with corresponding density level measurements (**B**). Immunohistochemistry of intra- and extra-cellular domains of TLR4 (brown) and nuclei counterstaining with hematoxylin (blue) (**C**). Values are mean±SEM (n = 4)/group *P<0.05 compared to SHAM.

### Cell Lysis

After completion of the mucin layer assay, cell media was removed and the cells were rinsed three times with PBS, scraped and lysed for Western blot analysis in RIPA buffer (Thermo Scientific, Rockford, IL) containing 1 mM proteinase (Sigma-Aldrich) and phosphatase (Thermo Scientific) inhibitors cocktail. Cells were transferred to Eppendorf tubes followed by incubation with agitation for 10 min at 4°C and centrifugation at 4°C for 10 min at 13,000 rpm. Total protein concentration in the supernatant was determined with the bicinchoninic acid protein assay.

### Paracellular Flux Measurement

The cells were seeded on 12-well Transwell plates (0.4-µm filter; Corning-Costar Corp, Cambridge, MA) to 100% confluency. FITC-dextran (20 kDa, 50 mg/ml, Sigma-Aldrich) in SFM with or without trypsin was added to the apical side of the epithelial cells with or without a mucin layer (see above). Cells were incubated for 60 min at 37°C, media from the bottom chamber was collected every 10 min and the amount of dextran in the basolateral side was measured (SpectraMax Gemini XS) at 492/520 nm (excitation/emission). Three independent experiments per group were performed.

### Western Blot

20 µg of protein of cell lysate or tissue homogenate were separated by SDS-PAGE. Membranes were incubated with primary antibodies as follows: mucin2, mucin13 and trypsin 1∶1000 (Santa Cruz Biotechnology), pancreatic amylase 1∶1000 (GeneTex, San Antonio, TX), intra- and extra-cellular domains of E-cadherin and TLR4 1:1000 (Abcam), intracellular domain of TLR4 1:1000 (Invitrogen). Secondary antibodies were diluted 1∶20000 (Santa Cruz Biotechnology) and detected with Super Signal West Pico (Thermo Scientific). The exposed x-ray films were scanned and label intensity was measured using digital gel analysis (NIH ImageJ software).

### Statistical Analysis

Results are presented as mean±SEM. Unpaired comparisons of mean values between groups were carried out by one-way ANOVA followed by Bonferroni post-hoc comparisons. P<0.05 was considered significant.

**Figure 8:**
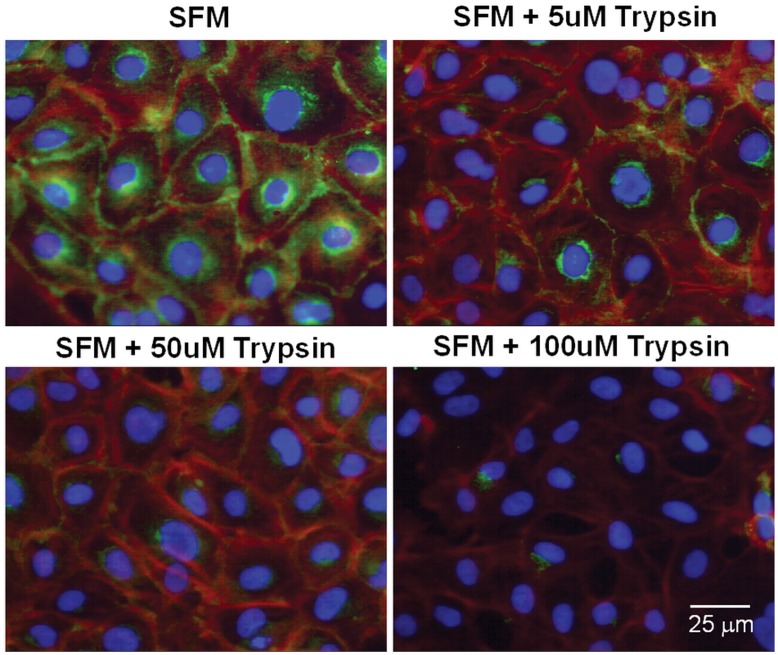
E-cadherin localization in intestinal epithelial cells after trypsin exposure. Representative micrographs of IEC-18 cells immunostained for the intra-cellular domain of E-cadherin (green), actin (red), and nuclei (blue) after exposure to serum free medium (SFM) with different trypsin concentrations.

**Figure 9:**
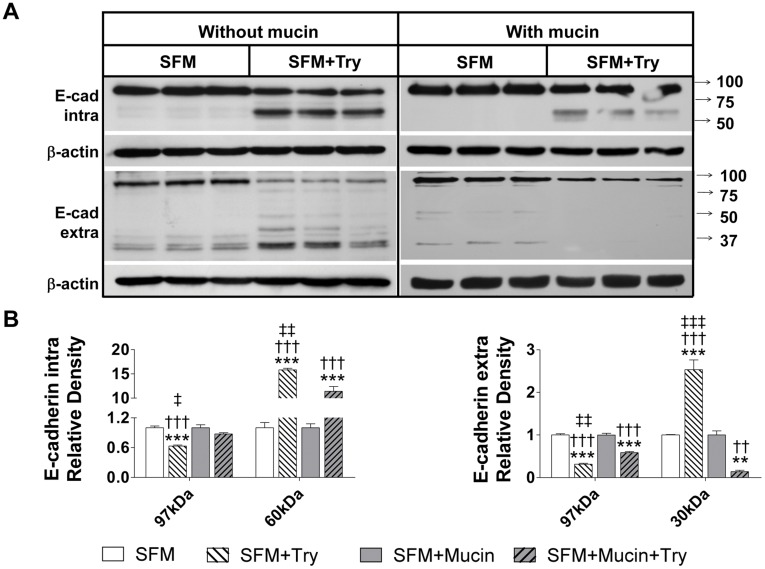
Mucin layer protects E-cadherin on intestinal epithelial cells from trypsin-mediated degradation. Western blot for intra- and extra-cellular domains of E-cadherin of IEC-18 cells without a mucin layer after exposure with serum free without and with trypsin (SFM and SFM+Try respectively) and IEC-18 cell with a mucin layer after exposure to serum free media with or without trypsin (SFM+Mucin and SFM+Mucin +Try) (**A**) with corresponding density levels measurements (**B**). Values are mean±SEM (n = 4)/group, **P<0.001 and ***P<0.0001 compared to SFM; ††P<0.001, †††P<0.0001 compared to SFM+Mucin; and ‡‡P<0.001, ‡‡‡P<0.0001 compared to SFM+Mucin+Try.

## Results

### Enzyme Activity in Intestinal Tissues

In order to determine the role of digestive enzymes in mucin disruption during ischemia, we used three inhibitors: acarbose, an α-glucosidase and pancreatic α-amylase inhibitor [25], tranexamic acid, a trypsin and plasminogen inhibitor [22], [26], [27]; and nafamostat mesilate, a broad spectrum serine protease inhibitor [28]). Acarbose inhibited amylase non-competitively with an inhibitory constant K_i_  = 0.48 mM and nafamostat mesilate inhibited trypsin competitively with K_i_  = 0.78 µM (Figure 1A and 1B). Tranexamic acid inhibited trypsin competitively with K_i_  = 56.5 mM [3].

**Figure 10:**
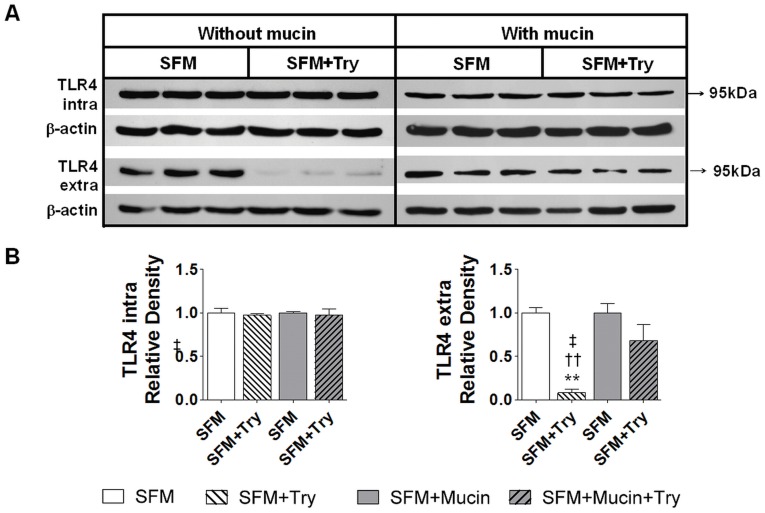
Mucin layer protects TLR4 on intestinal epithelial cells from trypsin-mediated degradation. Western blot for intra- and extra-cellular domains of E-cadherin of TLR4 cells without a mucin layer after exposure with serum free without and with trypsin (SFM and SFM+Try respectively) and IEC-18 cell with a mucin layer after exposure to serum free media with or without trypsin (SFM+Mucin and SFM+Mucin +Try) (**A**) with corresponding density levels measurements (**B**). Values are mean±SEM (n = 4)/group, **P<0.001 compared to SFM, ††P<0.001 compared to SFM+Mucin, and ‡P<0.05compared to SFM+Mucin+Try.

The activity levels of amylase and trypsin in intestine homogenates during ischemia were significantly higher compared to levels in sham animals (SHAM) and this activity was decreased in the groups treated with their respective inhibitors (Figures 2A, B). The activity of luminal contents (LUM CONT) alone in the sham group was significantly elevated as compared to the activity of tissue homogenates of all groups (Figures 2A, B). However, Western blot for amylase and trypsin in intestinal homogenates for all groups showed no change in the amount of protein, implying that the enzymes are being activated during ischemia (Figure 2C–E). In-situ zymography of trypsin activity in the intestinal wall (Figure 3) revealed no trypsin activity in the SHAM group and high levels of trypsin activity for the ischemic group without protease inhibitors (SAO30) and for the ischemic group with acarbose treatment (SAO30+ACA). Ischemic groups treated with tranexamic acid (SAO30+TA) and nafamostat mesilate (SAO30+ANGD) had undetectable levels of trypsin activity in the intestinal wall.

**Figure 11:**
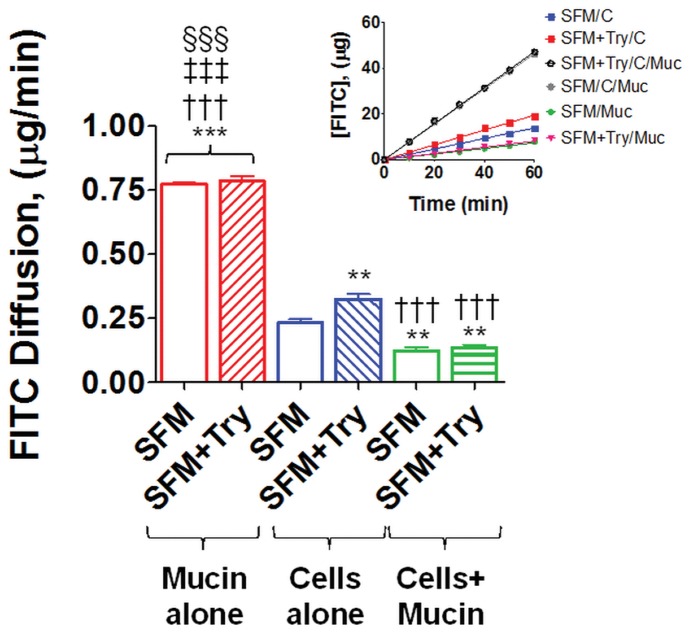
Mucin layer reduces FITC-dextran diffusion across the intestinal epithelial cells monolayer. Rate of diffusion of FITC-dextran across mucin alone, IEC-18 cell monolayer alone, or IEC-18 cell monolayer with a mucin layer after exposure to serum free media alone (SFM) or serum free media with trypsin (SFM+Try). Values are mean ± SEM (n = 4), **P<0.001 compared to SFM cells alone, †††P<0.001 compared to SFM+tryp cells alone, §§§P<0.0001 compared the other groups. (One-way Anova fallowed by Bonferroni’s post-hoc).

### Mucin 2 Degradation during SAO

To determine whether digestive enzymes mediate mucin degradation we visualized mucin 2 and trypsin with immunohistochemistry techniques. In the sham group (SHAM) mucin 2 had the appearance of an intact layer (Figure 4) while in the ischemic group without protease inhibitors (SAO30) this layer was discontinuous. The tips of the villi were destroyed and trypsin penetrated into the intestinal wall. In the presence of inhibitors the mucin 2 layer also appeared less dense as compared to the sham group. The protein levels of mucin 2 in intestine homogenates, as determined by Western blot (Figure 5A, B), were significantly decreased in the ischemic group as well as in the ischemic groups treated with tranexamic acid (SAO30+TA) and nafamostat mesilate (SAO30+ANGD). This evidence suggests that trypsin may not mediate mucin 2 degradation. Mucin 2 density in the ischemic group treated with acarbose (SAO30+ACA) was also decreased as compared to the sham group and was on average slightly greater than in the other ischemic groups (not significant).

In order to determine whether ischemia affects MUC2 mRNA synthesis we performed real-time quantitative PCR; the results show that mRNA relative levels in intestinal tissues were higher after 30 min of ischemia. mRNA levels in ischemic group without protease inhibition (SAO30) were significantly elevated compared to SHAM while the mRNA relative levels for the ischemic groups with luminal inhibitor treatments (SAO30+ACA, SAO30+TA, SAO30+ANGD) were not significantly increased compared to sham (Figure 5C).

### Mucin13 Fragmentation during SAO

Western blot analysis indicates that the digestive enzymes were not associated with a significant shift in the density levels of the intact molecule of mucin 13 (56 kDa) in any of the groups (Figure 5A, B).

Low molecular weight bands were observed in addition to the band corresponding to the whole molecule. The sham group (SHAM) had low molecular weight bands around 24 kDa and to lesser degree at 20 kDa. The density of these bands was significantly decreased in the ischemic group without luminal inhibitors (SAO30) and in the groups with trypsin inhibition by either tranexamic acid (SAO30+TA) or nafamostat mesilate (SAO30+ANGD) (Figure 5D). The ischemic group with amylase inhibition by acarbose (SAO30+ACA) had higher density levels of these bands. There was also another band around 17 kDa that was not present in the sham group and appeared during ischemia (Figure 5A). Density of this band was significantly greater in the ischemic group alone and in the groups with luminal inhibition by tranexamic acid or nafamostat mesilate compared to sham, and was present to a lesser degree in the acarbose group (not significant) (Figure 5D).

### Intestinal Ischemia Is Accompanied by E-cadherin Degradation and Increased Transport of FITC-dextran Into the Intestinal Wall

In order to determine whether mucin disruption during intestinal ischemia is accompanied by disruption of epithelial cell integrity we determined the levels of both intra- and extra-cellular domains of E-cadherin by Western blot (figure 6A, B). Both domains of E-cadherin were degraded after 30 min ischemia without protease inhibitor (SAO30). Inhibition of amylase (SAO30+ACA) and trypsin (SAO30+TA and SAO30+ANGD) yielded levels of E-cadherin that were higher than those in the ischemic group alone. However, these differences were not statistically significant.

FITC-dextran transport measurements show that in the sham group (SHAM) FITC-dextran remained intraluminal, lining the villi with negligible transfer from the lumen of the intestine into the intestinal wall (Figure 6C). In contrast, the ischemic groups without luminal inhibitors (SAO30) had erosion at the villi tips with observable FITC-dextran infiltration into the intestinal wall. Although the ischemic groups with enzyme inhibitors (SA030+ACA, SAO30+TA, SAO30+ANGD) had a preserved villus structure as seen on frozen sections, there was some penetration of FITC-dextran into the intestinal wall.

### TLR4 Is Degraded during Intestinal Ischemia

In order to determine whether mucin disruption is also accompanied by the degradation of typical membrane surface proteins, we determined the levels of the intra- and extra-cellular domains of TLR4 (Figure 7). Western blot analysis reveals that both domains of TLR4 were degraded after 30 min ischemia (SAO30) and this was not significantly reversed by inhibition of amylase (SAO30+ACA) and trypsin (SAO30+TA, SAO30+ANGD) (Figure 7A, B). Immunolabeling in the sham animals (SHAM) demonstrated both domains of TLR4 visible along the villi. In contrast, after 30 min ischemia without luminal inhibition of digestive enzymes (SAO30), visible morphological damage was accompanied by disruption of both domains of TLR4 (Figure 7C). Ischemic groups with luminal inhibition of amylase or trypsin (SA30+ACA, SAO30+TA, SAO30+ANGD) had preservation of the villus structure and greater staining than the ischemic group without inhibitors.

### Mucin Layer Reduces Trypsin-Mediated E-Cadherin and TLR4 Degradation in Intestinal Epithelial Cells *In Vitro*

In order to determine if trypsin disrupts the epithelial cells in a like manner to that observed in vivo in the rat model of intestinal ischemia and whether addition of a mucin layer prevents this, we studied an in vitro model using a monolayer of rat intestinal epithelial cells. After incubation of IEC-18 cells for 1 hour with 5 µM trypsin, E-cadherin immunohistochemical labeling was decreased without alteration in cell shape or the monolayer (Figure 8). At higher trypsin concentrations, the epithelial actin cytoskeleton started to disappear, the cells retracted and the monolayer began to disintegrate. Western blot for both intra- and extra-cellular domains of E-cadherin revealed degradation of this protein in whole-cell lysates after one hour incubation with serum free media containing 5 µM trypsin (SFM+Try) (Figure 9). This is supported by reduced density of the whole molecule as seen with antibodies against both intra- and extra-cellular domains as well as the appearance of lower molecular weight bands. Application of a layer of 10% mucin on the apical side of the cells prior to the addition of the serum free media with trypsin resulted in higher density of the whole molecule of both intra- and extra-cellular domains of E-cadherin and decreased density of their corresponding lower molecular weight bands (Figure 9). Similarly, density levels for the extracellular domain of TLR4 were reduced in intestinal epithelial cells that were treated with serum free media containing trypsin (SFM+Try) as compared to those without trypsin treatment (SFM) (Figure 10). A mucin layer on the apical side of the cells reduced degradation of the TLR4 extra-cellular domain. Density levels of the intra-cellular domain of TLR4 were unchanged in all groups.

### Mucin Layer Reduces Trypsin-Mediated FITC-dextran Transport

In order to assess the ability of the mucin layer to act as a barrier against trypsin-mediated epithelial cell permeability increases, we measured the rate at which FITC-dextran (20 kDa) diffused across the epithelial cell monolayer (Figure 11). The addition of 5 µM trypsin in serum free media (SFM+Tryp) to the apical side of the IEC-18 cell monolayer resulted in significantly increased FITC-dextran levels on the basolateral side of the cell monolayer as compared to cells without apical trypsin (SFM). When a mucin layer was added on the apical side prior to addition of serum free media with or without trypsin, the levels of FITC-dextran measured on the basolateral side were not significantly different between the groups. In addition, diffusion of FITC-dextran across the epithelial cell monolayer in cultures not exposed to protease but containing a mucin layer was significantly reduced as compared to cells that did not have a mucin layer.

## Discussion

The importance of mucin in the breakdown of the intestinal mucosal function in ischemia and hemorrhagic shock was first suggested in the 1960s [29]. Since then a number of investigators have established a correlation between the loss of the intestinal mucus layer and increased intestinal injury during hemorrhagic shock [4] and sepsis [30], [31]. In a recent study we showed that 30 minutes of intestinal ischemia results in mucin disruption and transport of digestive enzymes from the lumen of the intestine into the intestinal wall and subsequently the systemic circulation [22]. We also showed that among the serine proteases, trypsin appears to play an important role in the injury observed during intestinal ischemia [22].

In this study we demonstrated *in vivo* with ischemic bowel and endogenous digestive enzymes in the intestinal lumen, as well as *in vitro* using intestinal epithelial cell cultures and trypsin in the culture media, that degradation or absence of mucin results in loss of epithelial function as a barrier. In both cases we observed loss of E-cadherin, increased permeability to FITC-dextran, and degradation of TLR4. Trypsin concentration in the rat intestinal lumen has been reported to be between 1 to 40 µM [32], [33]; the typical trypsin concentration used to pass cells in cell culture is 0.5 g/L (22 µM) [34]. In the present studies, we show that incubation of intestinal epithelial cells with 5 µM trypsin for one hour is sufficient to degrade E-cadherin and TLR4 without destroying the monolayer (Figure 8). We demonstrated in vivo that sham animals have an intact mucin layer with preserved epithelial cell integrity and function; similarly we showed in vitro that having a mucin layer on the apical side of the cell monolayer protects the intestinal epithelial cells against trypsin-mediated disruption (Figures 9 and10). Collectively these results suggest that if digestive enzymes such as trypsin are given the opportunity to come in contact with enterocytes at concentrations normally found in the intestinal lumen the result will be loss of integrity and function of the epithelial barrier.

An intact mucin layer in normal intestine is then important to protect epithelial membrane proteins against enzymatic attack; for instance, degradation of E-cadherin junctions results in increased epithelial permeability. Degradation of membrane receptors, such as TLR4, disrupts the usual pathway for TLR4 signaling after binding of lipopolysaccharides after as little as 30 min of ischemia. It remains to be investigated whether degradation of the extracellular domain of TLR4 may in itself initiate a signaling cascade, as has been reported for TLR5 and TLR15 [35], [36].

In this study we also showed in vivo that mucin disruption during early periods of intestinal ischemia is not directly mediated by trypsin. Mucin 2 and mucin 13 are degraded during ischemia but this disruption is not prevented by the inhibition of trypsin with tranexamic acid or nafamostat mesilate (Figure 5). There is some preservation of mucin 2 and mucin13 by amylase inhibition with acarbose, which may suggest an indirect role of pancreatic amylase; the role of amylase remains to be further investigated. Furthermore the ischemic groups treated with luminal injection of enzyme inhibitors had reduced enzymatic activity and these groups had less visible injury as compared to the ischemic group without inhibitors. These results suggest that individual digestive enzymes or a combination of them mediate epithelial cell disruption but not mucin disruption.

Goblet cell-derived mucin (mucin 2) in the mucus layer has been proposed to be the major barrier against passage of high-molecular weight compounds across the intestinal barrier [37]. The level of the mucus layer is determined by a balance between secretion and degradation of this protein. A variety of stimuli in different pathologies may lead to mucin over-secretion or cessation of secretion [38], [39], [40], [41], [42]. In the current results we show that after 30 min ischemia the level of MUC2 mRNA is significantly increased as compared to the sham group. This rapid response after a relatively short period of ischemia may serve to replenish mucin 2 in the mucus layer, which may be a critical protection mechanism to prevent the escape of luminal digestive enzymes. Further studies are necessary to determine whether MUC2 mRNA is translated if longer periods of ischemia and reperfusion are present and if under these conditions the rate of mucin degradation is greater than the rate of secretion, resulting in irreversible injury.

While the importance of the epithelial barrier in intestinal homeostasis is well recognized, the significance of the mucus layer with regard to passage of digestive enzymes remains less certain. We show here *in vitro* that addition of an exogenous layer of mucin to the apical side of intestinal epithelial cells significantly reduces trypsin-mediated disruption of E-cadherin and TLR4 but does not completely prevent it. One explanation could be that the exogenous mucin as used in the current experiment is of gastric origin, which contains MUC5 instead of MUC2. Although from different origins these two mucin isoforms have similar general characteristics [43], [44]. In addition, it may be that complete protection against trypsin degradation by an exogenous mucin layer on an epithelial monolayer is not achieved because the mucin used in this experiment is partially purified and may not form a complete barrier as it is found *in vivo*.

In this study we investigate how two different mucin isoforms found in the small intestine (mucin2 and mucin13) are disrupted after splanchnic ischemia. However we cannot rule out the possible role of other mucin isoforms that although present in soluble form do not comprise a major part of the mucosal barrier; for instance mucin4, which is localized in the paneth cells but not in goblet cells of the small intestine [45] or mucin1 or mucin3 different types of membrane bound mucin. Functional studies to determine the role of mucin2 or mucin13 during intestinal ischemic injury present some challenges. Up to date only a knockout mice model for mucin2 gene (MUC2) is available. This model was first described in 2002 [46] and since then the effect of mucin2 deficiency has been largely characterized for the large intestine but less is known in this regard about the small intestine. The MUC2^−/−^ mice have significantly lower weight at 5 weeks after weaning (12 g as compared to 20 g for their wild type litter mates) [47]. MUC2^−/−^ mice also develop colitis as early as 5 weeks manifest in form of diarrhea, gross bleeding and lethargic state [46], [47]. MUC2^−/−^ mice have goblet cells with abnormal morphology, decreased epithelial barrier function, and altered expression in tight junction-related genes such as upregulation of claudin-10 and downregulation of claudin1 and claudin 5 [46], [48]. In addition there is upregulation of genes stimulating cell growth which manifests in intestinal tumor formation with spontaneous progression to invasive carcinoma later in life [46]. Due to the fact that MUC2^−/−^ mice do not have normal intestinal function after weaning which is usually the time used in experimental studies, MUC2^−/−^ mice are not appropriate to study the breakdown of the mucosal epithelial barrier because the barrier is already altered in this model.

In conclusion, the results presented here confirm that mucin in the mucus layer is a barrier that protects the intestinal epithelium against enzymatic auto-digestion. During ischemia mucin isoforms are degraded, which may stimulate synthesis of new mucin to restore the damaged barrier. As mucin is degraded and the barrier function of the mucus layer is thus compromised, digestive enzymes in the lumen of the intestine are allowed to come into contact with the epithelium with consequent loss of epithelial cell integrity and function. This cascade of events results in intestinal injury, transport of digestive enzymes into the systemic circulation and subsequent shock.
